# Synergistic cytotoxicity of cisplatin and Taxol in overcoming Taxol resistance through the inhibition of LDHA in oral squamous cell carcinoma

**DOI:** 10.3892/ol.2015.2931

**Published:** 2015-02-03

**Authors:** LIN FENG, LING-LING E, MICHAIL MICHAILOVICH SOLOVEIV, DONG-SHENG WANG, BO ZHANG, YU WAN DONG, HONG-CHEN LIU

**Affiliations:** 1 Institute of Stomatology, Chinese People’s Liberation Army General Hospital, Beijing 100853, P.R. China; 2 Oral Medical Research Center, Saint-Petersburg Medical University, Saint-Petersburg 197101, Russia

**Keywords:** Taxol resistance, oral squamous cell carcinoma, cisplatin resistance, glucose metabolism

## Abstract

The development of chemoresistance in patients represents a major challenge in cancer treatment. Lactate dehydrogenase-A (LDHA) is one of the principle isoforms of LDH that is expressed in breast tissue, controlling the conversion of pyruvate to lactate and also playing a significant role in the metabolism of glucose. The aim of this study was to identify whether LDHA was involved in oral cancer cell resistance to Taxol and whether the downregulation of LDHA, as a result of cisplatin treatment, may overcome Taxol resistance in human oral squamous cells. The OECM-1 oral epidermal carcinoma cell line was used, which has been widely used as a model of oral cancer in previous studies. The role of LDHA in Taxol and cisplatin resistance were investigated and the synergistic cytotoxicity of cisplatin and/or Taxol in oral squamous cells was analyzed. Cell viability was analyzed by MTT assay, LDHA expression was analyzed by western blot analysis and siRNA tranfection was performed to knock down LDHA expression. The present study results showed that decreased levels of LDHA were responsible for the resistance of oral cancer cells to cisplatin (CDDP). CDDP treatments downregulated LDHA expression, and lower levels of LDHA were detected in the CDDP-resistant oral cancer cells compared with the CDDP-sensitive cells. By contrast, the Taxol-resistant cancer cells showed elevated LDHA expression levels. In addition, small interfering RNA-knockdown of LDHA sensitized the cells to Taxol, but desensitized them to CDDP treatment, while exogenous expression of LDHA sensitized the cells to CDDP, but desensitized them to Taxol. The present study also revealed the synergistic cytotoxicity of CDDP and Taxol for killing oral cancer cells through the inhibition of LDHA. This study highlights LDHA as a novel therapeutic target for overcoming Taxol resistance in oral cancer patients using the combined treatments of Taxol and CDDP.

## Introduction

Taxol (paclitaxel) is a valuable cancer chemotherapeutic agent used for the treatment of numerous types of cancer, including ovary, breast, oral and lung carcinomas (1–4). The primary cellular targets of Taxol are the microtubules of cancer cells, which are vital for mitotic activity, cellular motility and proliferative capacity (3). Taxol is also known to block cell replication, arrest cells in the G_2_/M phase of the cell cycle and induce apoptosis (5,6). Despite significant clinical responses initially, the majority of patients eventually develop resistance to Taxol. Currently, mechanisms accounting for Taxol resistance include alterations to the tubulin structure (7–9), changes in the drug-binding affinity of the microtubules (10) and cell cycle deregulation (11). However, the detailed molecular mechanisms that may contribute to the Taxol resistance of cancer cells are not fully understood.

Cisplatin (CDDP) is a DNA-damaging agent that induces cytotoxicity through the production of DNA damage caused by the formation of CDDP-DNA adducts (12), which leads to irreparable DNA damage and ultimately, cell death. However, similar to Taxol, the development of CDDP resistance in cancer cells is a major impediment in clinical treatment (13). Currently, the mechanisms of CDDP resistance remain unclear. It has been reported that CDDP and other anticancer agents induce the activation of the epidermal growth factor receptor in multiple cancer cells that overexpress the receptor (14). In addition, another study described the fact that the uptake of various compounds, including nutrients such as glucose, was reduced in CDDP-resistant hepatoma cells compared with CDDP-sensitive cells (15). Moreover, a recent study reported that the knockdown of GLUT-1, which is a glucose transporter on the cell membrane, facilitated CDDP treatment, resulting in increased rates of apoptosis in oral cancer cells under hypoxic conditions (16), indicating that the glucose metabolism pathway is involved in CDDP resistance.

Lactate dehydrogenase-A (LDHA) is one of the principle isoforms of LDH that is expressed in breast tissue. LDHA controls the conversion of pyruvate to lactate and plays a significant role in glucose metabolism (17). A study has also shown that LDHA is important in Taxol resistant breast cancer cells (18). This study detected increased LDHA expression and activity in Taxol-resistant cells, and identified that the sensitivity of Taxol-resistant cells to Taxol was significantly increased by the downregulation of LDHA by small interfering (si)RNA, indicating that LDHA may be a therapeutic target for overcoming Taxol resistance.

As previous studies have shown that CDDP treatment inhibits LDHA expression in lung cancer cells (15), in the present study investigated whether LDHA is downregulated by CDDP treatments in oral squamous cancer cells. In addition, the role of LDHA in Taxol resistance was investigated. Whether synergistic cytotoxicity of cisplatin and Taxol on Taxol resistant oral cancer cells occurs via the inhibition of LDHA was also explored. This study will provide a theoretical explanation to support the combined treatment of Taxol and CDDP to develop clinical chemotherapeutic strategies for oral cancer patients.

## Materials and methods

### Cell culture and conditions

The human oral squamous cell carcinoma (OSCC) cell lines, OECM-1 and H-1, were purchased from the American Type Culture Collection (Manassas, VA, USA). The cultivation conditions were used as described previously (19). Briefly, the cells were routinely cultured in Dulbecco’s modified Eagle’s medium (DMEM; Gibco BRL, Paisley, UK) containing 10% fetal bovine serum (FBS; HyClone, Logan, UT, USA), at 37°C in a humid atmosphere with 5% CO_2_.

### Cell viability assay

The cancer cells were treated with Taxol, 5-fluorouracil (FU) or CDDP with the indicated concentrations for 24 h. The cells were seeded in a 96-well plate, at a density of 3,000 cells/well in 0.2 ml DMEM containing 10% FBS. Following overnight incubation under the same cultivating conditions, each well was refreshed with 0.2 ml serum-free medium (SFM) for another day. The cells were then treated with 0.2 ml SFM containing various concentrations of Taxol, CDDP, 5-FU, Taxol/CDDP or Taxol/5-FU. The drug-containing SFM was refreshed after 2 days and incubated under the same conditions for another 2 days. Finally, cell viability was accessed with an MTT reagent (Sigma-Aldrich, Inc., St. Louis, MO, USA), and by measuring the absorbance at 590 nm with a plate reader. Relative viability was obtained from the absorbance at 590 nm of the drug-treated OECM-1 cells divided by the absorbance at 590 nm of the untreated OECM-1 cells. The same experiment was repeated three times.

### Western blotting and antibodies

The cells were harvested and lysed in a buffer containing 50 mM Tris-HCl (pH 7.5), 150 mM NaCl, 2 mM EDTA, 1% Triton, 1 mM PMSF and a protease inhibitor cocktail (Sigma-Aldrich) for 20 min on ice. Lysates were separated by centrifugation at 16,000 × g, at 4°C for 10 min. Supernatants were collected and protein concentrations were determined by the Bradford assay (Bio-Rad, Hercules, CA, USA). The proteins were then separated with an SDS/polyacrylamide gel and transferred to a nitrocellulose membrane (Bio-Rad). Subsequent to being blocked in phosphate-buffered saline (PBS) with 5% skimmed dry milk for 1 h, the membranes were incubated overnight at 4–8°C with the primary antibodies in PBS with 5% skimmed dry milk. The following antibodies were utilized: Anti-LDHA rabbit antibody (1:1,000; Cell Signaling Technology, Inc., Danvers, MA, USA) and anti-β-actin monoclonal antibody (1:2,000; Sigma-Aldrich). Membranes were extensively washed with PBS and incubated with horseradish peroxidase conjugated secondary anti-mouse antibody or anti-rabbit antibody (1:2,000; Bio-Rad). Subsequent to additional washes with PBS, antigen-antibody complexes were visualized with an enhanced chemiluminescence kit (Pierce Biotechnology, Inc., Rockford, IL, USA).

### Generation of Taxol- and CDDP-resistant cell lines

The Taxol- and CDDP-resistant cell lines were generated according to previously described methods (18,19). Briefly, OECM-1 Taxol-resistant (Tax R) or (Cis R) CDDP-resistant cells were developed from parental OECM-1 cells by treating them with gradually increasing concentrations of Taxol or CDDP in regular cell culture medium. Tax R or Cis R single or pooled clones were identified and cultured. All resistant cells were verified by the treatments every four weeks.

### siRNA and plasmid DNA transfection

siRNA oligonucleotides for LDHA were purchased from Sigma-Aldrich, with a scrambled siRNA (Sigma-Aldrich) used as a control. A vector containing wild-type LDHA was purchased from Origene (RC209378; Rockville, MD, USA). Transfection was performed using the Oligofectamine Transfection Reagent (Invitrogen Life Technologies, Carlsbad, CA, USA) according to the manufacturer’s protocol. At 48 h post-transfection, whole-cell lysates were prepared for further analysis.

### Statistical analysis

The unpaired Student’s t-test was used for the data analysis. All data are presented as the mean ± standard error. P<0.05 was considered to indicate a statistically significant difference.

## Results

### LDHA is downregulated in response to CDDP treatment

**Figure 1:**
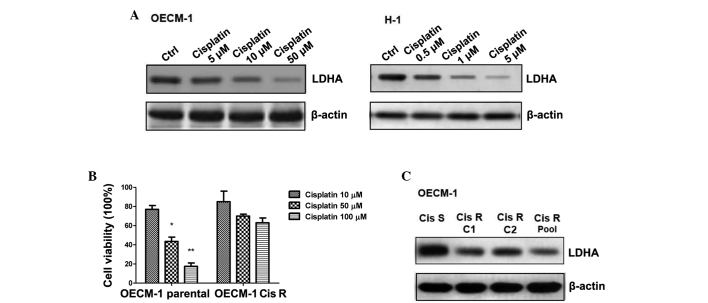
Cisplatin (CDDP) treatments decrease lactate dehydrogenase-A (LDHA) expression. (A) CDDP treatments at 5, 10 and 50 μM in OECM-1 and H-1 cells decreased the level of LDHA expression. β-actin served as a loading control. (B) Generation of the CDDP-resistant cell line. OECM-1 CDDP-sensitive (Cis S) and CDDP-resistant (Cis R) cells were treated at 10, 50 and 100 μM CDDP, followed by the measurement of cell viability. (C) Cis R clone number 1 (C1), and Cis R clone number 2 (C2) and the Cis R pool were cultured, and western blotting was performed to examine the LDHA expression levels. β-actin served as a loading control. The data in the columns represents the mean of three independent experiments and the bars represent the standard error. ^*^P<0.05 and ^**^P<0.01 vs. control.

The present study investigated which signaling pathway may be involved in CDDP resistance, according to LDHA expression. Following screening, which involved the investigation of mitochondrial oxidation consumption, the AKT and mTOR pathway and microRNAs which may target glycolysis and mitochondrial apoptosis pathways, including cytochrome *c* and Bcl-2, the expression of LDHA was found to be significantly decreased subsequent to CDDP treatment at varying doses in two oral cancer cell lines (P<0.05; Fig. 1A). Next, a CDDP-resistance cell line was generated. The OECM-1 cells, which have been widely used as a model of oral cancer in previous studies, were treated with gradually increasing concentrations of CDDP in cell culture medium for the selection of resistant cells. Following successive treatments for 3 months, several resistant cell clones were developed (Fig. 1B). More of the Cis R cells remained alive following the addition of 50 and 100 μM CDDP, while the OECM-1-sensitive (Cis S) cells exhibited significant cell death following the 50- and 100-μM treatments (P<0.05 and P<0.01, respectively). We hypothesized that the expression of LDHA should be altered in the Cis R cells, and the data in Fig. 1C confirmed this by showing that the level of LDHA expression in the Cis R cells was decreased. Taken together, these results revealed a tight correlation between the expression of LDHA and CDDP resistance.

### Tax R cells display upregulated LDHA expression

**Figure 2:**
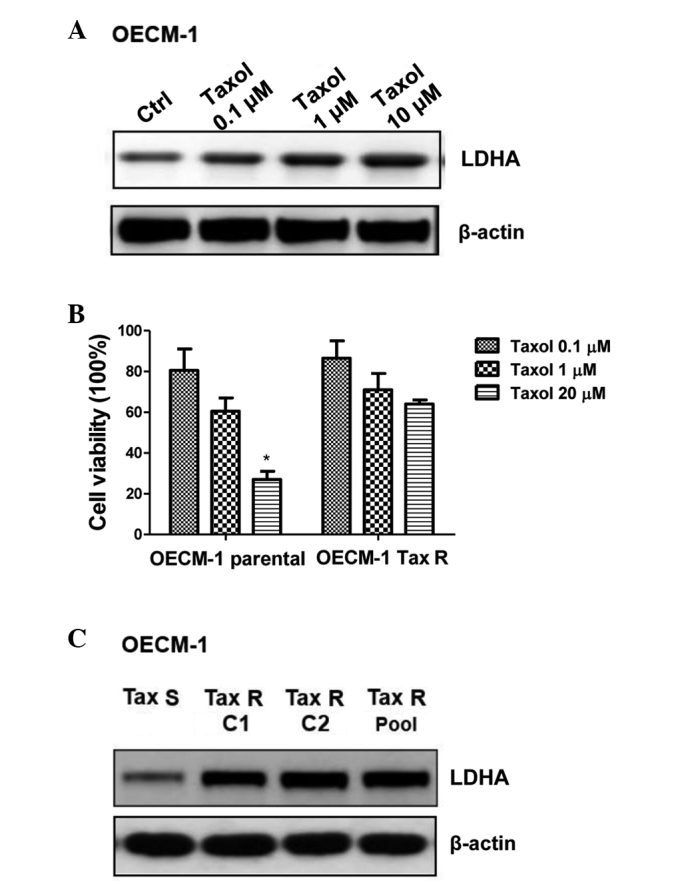
Taxol-resistant cells shows increased lactate dehydrogenase-A (LDHA) expression. (A) Taxol treatments of 0.1, 1 and 10 μM in the OECM-1 cells induced LDHA expression. β-actin served as a loading control. (B) Generation of the Taxol-resistant cell line. OECM-1 Taxol-sensitive (Tax S) and Taxol-resistant (Tax R) cells were treated with 0.1, 1 and 20 μM Taxol, followed by the measurement of cell viability. (C) Tax R clone number 1 (C1), Tax R clone number 2 (C2) and the Tax R pool were cultured, and western blotting was performed to examine the LDHA expression levels. β-actin served as a loading control. The data in the columns represent the mean of three independent experiments, and the bars represent the standard error. ^*^P<0.05 vs. control.

It has been reported that LDHA has a significant role in Taxol-resistant breast cancer cells (18). The present study next checked the LDHA expression levels in response to Taxol treatment in the oral cancer cells. Fig. 2A shows that Taxol treatment at varying concentrations induced LDHA expression. Similar to the generation of CDDP resistance cell line, a Taxol-resistant cell line was generated for OECM-1. More of the OECM-1 Taxol-resistant (Tax R) cells remained alive following the addition of 1 and 20 μM CDDP, while the OECM-1 Taxol-sensitive (Tax S) cells exhibited significant cell death following the 1- and 20-μM treatments (Fig. 2B; P<0.05). As expected, the LDHA expression level was upregulated in the Tax R cells, indicating that LDHA is an important glycolytic enzyme involved in Taxol-resistance (Fig. 2C).

### Downregulation of LDHA re-sensitizes oral cancer cells to Taxol

**Figure 3:**
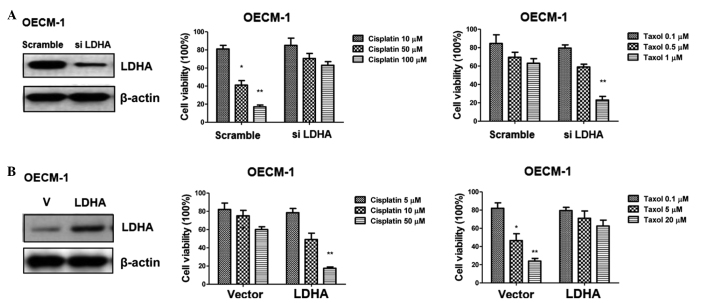
Lactate dehydrogenase-A (LDHA) plays reverse roles in response to Taxol and cisplatin (CDDP) treatments. (A) Knockdown of LHDA sensitized the OECM-1 cells to Taxol treatment, but desensitized the OECM-1 cells to the CDDP treatment. The OECM-1 cells were transfected with small interfering (si)RNA for 48 h, then treated with CDDP at 10, 50 and 100 μM or Taxol at 0.1, 0.5 and 1 μM, followed by the measurement of cell viability. (B) Overexpression of LDHA sensitized the OECM-1 cells to CDDP treatment, but desensitized the OECM-1 cells to Taxol treatment. The OECM-1 cells were transfected with overexpression vector containing wild-type LDHA (V) for 48 h, then treated with CDDP at 5, 10 and 50 μM or Taxol at 0.1, 5 and 20 μM, followed by the measurement of cell viability. The columns represent the mean of three independent experiments, and the bars represent the standard error. ^*^P<0.05 and ^**^P<0.01 vs. control.

To further support the results, LDHA-knockdown was performed using siRNA specific to LDHA in the OECM-1 cells, followed by the measurement of the cell sensitivities to the Taxol and CDDP treatments. Fig. 3A shows the efficient knockdown of LDHA, which rendered the oral cells insensitive to CDDP treatment, but sensitive to Taxol treatment.

### Upregulation of LDHA re-sensitizes oral cancer cells to CDDP

The results were then verified by the overexpression of LDHA by transient transfection of a vector containing wild-type LDHA. The OECM-1 cells consistently exhibited a higher level of LDHA and an acquired resistance to Taxol, but became vulnerable to CDDP (Fig. 3B). Taken together, these data showed that LDHA plays reverse roles in the response to Taxol and CDDP.

### Combination of Taxol and CDDP shows a synergistic effect on Tax R cells by blocking LDHA expression

**Figure 4:**
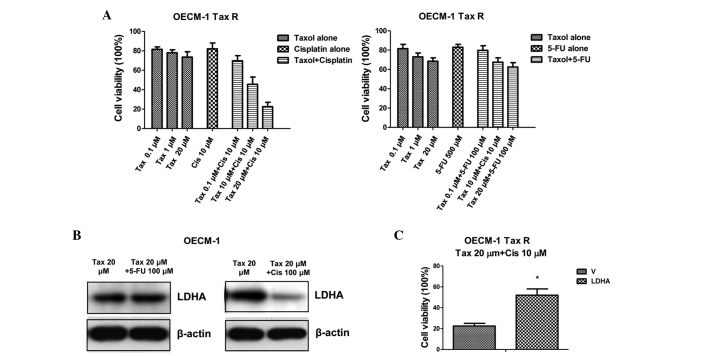
Combination of Taxol and cisplatin (CDDP) shows synergistic effects on the Taxol-resistant (Tax R) cells through the inhibition of lactate dehydrogenase-A (LDHA). (A) Tax R cells were treated with Taxol alone at 0.1, 1 and 20 μM, CDDP alone at 10 μM, 5-FU alone at 500μM, Taxol plus CDDP or Taxol plus 5-fluorouracil (FU), followed by the measurement of cell viability. (B) LDHA was downregulated in response to the treatment with the combination of Taxol and CDDP, but exhibited no change following treatment with the combination of Taxol and 5-FU. (C) The overexpression of LDHA in the Taxol-resistant (Tax R) cells resulted in acquired resistance to treatment with the combination of Taxol and CDDP. The cells were transfected with a vector containing wild-type LDHA (V) for 48 h, followed by depletion of the medium and treatment with the drugs for 24 h. A cell viability assay was performed. Columns represent the mean of three independent experiments, and bars represent the standard error. ^*^P<0.05 vs. control.

Since it has been reported that the combination of Taxol with CDDP treatment shows synergistic cytotoxicity in OECM-1 cells (19). In the present study, experiments were designed to examine whether treating Taxol-resistant oral cancer cells with CDDP would result in synergistically therapeutic effects. Fig. 4A shows that treatment with Taxol or CDDP alone in the Tax R cells exhibited no significant inhibitory effects, but that combining Taxol and CDDP together resulted in significant inhibition of cell viability (Fig. 4A; P<0.05). Since it has been reported that 5-FU-resistant cancer cells exhibit upregulated glucose metabolism (20), the present study then treated the Tax R cells with a combination of Taxol and 5-FU. The results showed that the combination of Taxol and 5-FU did not generate better chemotherapeutic effects (Fig. 4A), indicating that the synergistic inhibitory effects of Taxol plus CDDP acted through the inhibition of the expression of LDHA by CDDP in the Tax R cells. To support this conclusion, the LDHA expression was compared for the combined treatments; the LDHA expression level was significantly downregulated by Taxol and CDDP (P<0.05), but no change was evident following treatment with Taxol and 5-FU (Fig. 4B). When LDHA was exogenously overexpressed, the OECM-1 parental cells obtained resistance to the combined treatments (Fig. 4C), indicating that the downregulation of LDHA specifically accounted for the mechanisms of the synergistic effects of the combination of Taxol and CDDP. In summary, these results suggested that CDDP plays essential roles in overcoming Taxol resistance in oral cancer cells.

## Discussion

CDDP is employed for the treatment of a wide array of solid malignancies via multiple mechanisms, such as those that involve the steps prior to CDDP binding to DNA (pre-target resistance), those directly associating DNA-CDDP adducts (on-target resistance), those associated with the lethal signaling pathway(s) induced by CDDP-mediated DNA damage (post-target resistance) and those that affect molecular circuitry without clear links to CDDP-induced signals (off-target resistance) (13). LDH-A is one of the main isoforms of LDH expressed in breast tissue, catalyzing the conversion of pyruvate to lactate, which is a key step in glucose metabolism. It has previously been shown that LDH-A plays a vital role in glycolysis, growth properties and tumor maintenance, as well as in the chemoresistance of breast cancer cells (18). To date, few studies have focused on the association between glucose metabolism and CDDP resistance in oral cancer cells. However, it has been shown that CDDP-resistant cancer cells commonly exhibit slower proliferation rates and reduced uptake levels of various compounds, including nutrients (15). Another study has reported the function of GLUT-1 in CDDP resistance. This study reported the silencing of GLUT-1-sensitized oral cancer cells to CDDP during hypoxia (16), indicating that CDDP-resistant cells had an impaired glucose metabolism pathway.

Taxol is a widely used chemotherapeutic agent for the treatment of several types of cancer, including oral cancer. Taxol resistance may result in the subsequent recurrence and metastasis of cancer. The specific mechanisms involved remain poorly understood, although extensive investigations have been conducted. It has been reported that treatment with the combination of Taxol and CDDP in human OSCC shows a synergistic effect (19), although the mechanisms remain unclear. In the present study, it was found that CDDP treatment resulted in decreased LDHA expression levels in the oral cancer cells, while Taxol treatment showed the reverse results, with an increased level of LDHA expression. In addition, Taxol-resistant cells showed increased LDHA expression and the CDDP-resistant cells showed decreased LDHA expression.

The downregulation of LDHA by LDHA siRNA increased the sensitivity of the cells to Taxol, but increased their resistance to CDDP. This indicated that Taxol treatment triggers the glucose metabolism pathway to ensure cancer cell survival, most likely through promoting cell glycolysis. A previous study has shown that cancer cells inhibit cytochrome *c*-mediated apoptosis by a mechanism of deregulated glucose metabolism (21). Thus, the Taxol-induced high expression levels and activity of LDHA detected in Taxol-resistant cells could be an adaptation of these cells to Taxol treatment and may be used to modulate glucose metabolism and glycolysis to avoid the apoptosis induced by Taxol. As aforementioned, CDDP showed a high efficiency in the inhibition of LDHA, which may result in the further examination of the synergistic effects of the combination of these two drugs. As expected, CDDP re-sensitized the Tax R cells through the inhibition of LDHA, and this effect was reversed following overexpression of LDHA in the parental oral cancer cells. Our future studies will continue to investigate the therapeutic effects on the recovery of chemoresistance through the alteration of glucose metabolism by specific glycolysis inhibitors. Taken together, the results of this study indicate that LDH may potentially serve as a target for overcoming Taxol resistance in human breast cancer patients.
